# Bioremediation of Polycyclic Aromatic Hydrocarbons by Means of Bacteria and Bacterial Enzymes

**DOI:** 10.3390/microorganisms12091814

**Published:** 2024-09-02

**Authors:** Anastasiia T. Davletgildeeva, Nikita A. Kuznetsov

**Affiliations:** 1 Institute of Chemical Biology and Fundamental Medicine, Siberian Branch of Russian Academy of Sciences, Novosibirsk 630090, Russia; nikita.kuznetsov@niboch.nsc.ru; 2 Department of Natural Sciences, Novosibirsk State University, Novosibirsk 630090, Russia

**Keywords:** polycyclic aromatic hydrocarbons, pollution, toxicity, remediation, bacterial bioremediation, enzymatic degradation

## Abstract

Polycyclic aromatic hydrocarbons (PAHs) are widespread, persistent, and toxic environmental pollutants. Many anthropogenic and some natural factors contribute to the spread and accumulation of PAHs in aquatic and soil systems. The effective and environmentally friendly remediation of these chemical compounds is an important and challenging problem that has kept scientists busy over the last few decades. This review briefly summarizes data on the main sources of PAHs, their toxicity to living organisms, and physical and chemical approaches to the remediation of PAHs. The basic idea behind existing approaches to the bioremediation of PAHs is outlined with an emphasis on a detailed description of the use of bacterial strains as individual isolates, consortia, or cell-free enzymatic agents.

## 1. Introduction

Population growth, global industrialization, constantly increasing energy demand, and the widespread use of pesticides cause more and more various pollutants to enter aquatic and soil ecosystems and accumulate there [1,2,3,4]. In the modern world, the main source of energy for industrial and domestic needs is products derived from petroleum [5]. Meanwhile, during the prospecting, extraction, processing, transportation, and storage of petroleum products, spills and leaks of petroleum hydrocarbons into the environment inevitably take place [6,7]. Petroleum-related pollution of soils diminishes soil strength and permeability, increases dryness, and changes the moisture content, thereby ultimately leading to a reduced yield of crops [8]. In this context, soil pollution is considered significant already when the concentration of pollutants reaches 1% (*w*/*w*) [3]. The presence of various pollutants in water, such as oils, heavy metals, and organic dyes and solvents, limits the world’s sources of clean water and overall has an adverse impact on aquatic ecosystems [9,10]. The ubiquitous spread of environmental pollution and proven negative consequences of such pollution for humans and other living organisms have prompted research into possible approaches to pollutant degradation and the restoration of the environment.

**Figure 1:**
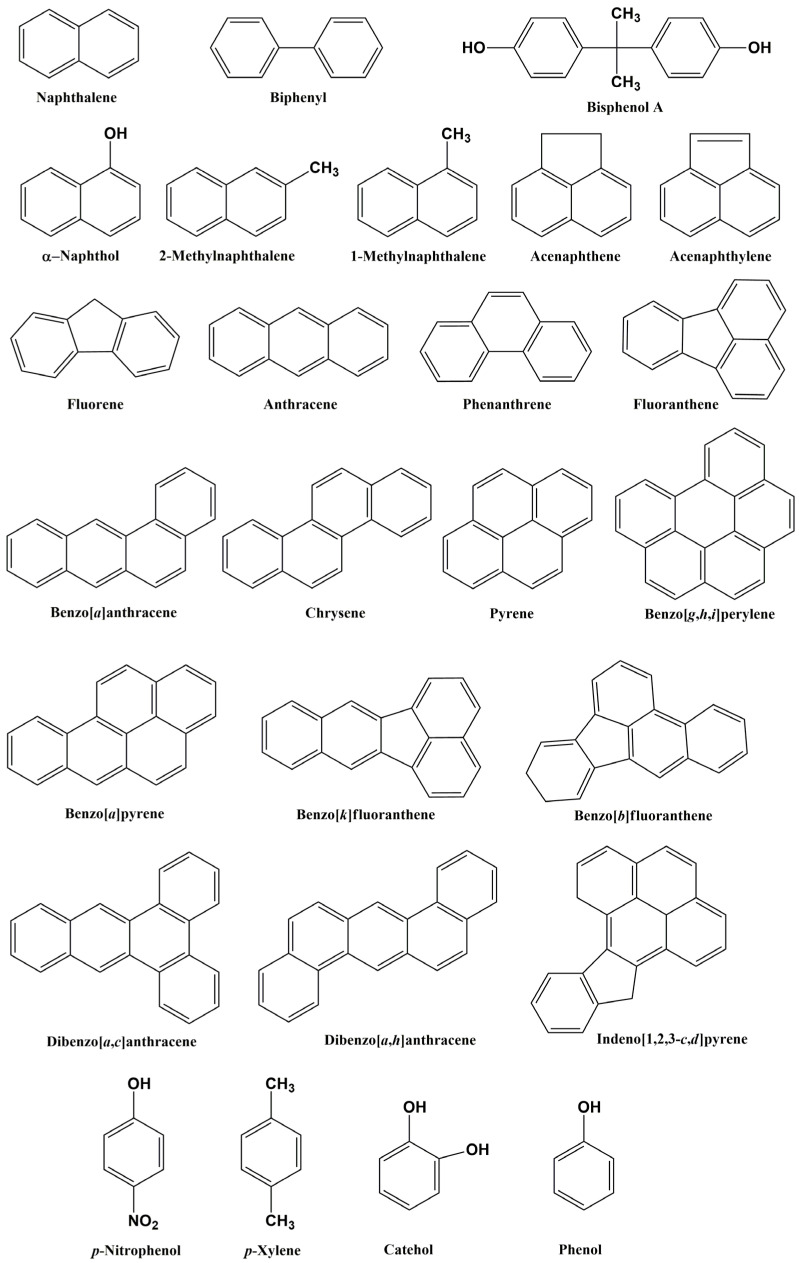
Chemical structures of the most commonly analyzed PAHs and some of their derivatives.

Xenobiotic aliphatic and aromatic compounds derived from petroleum hydrocarbons have become major environmental pollutants lately [11]. Among such substances, polycyclic aromatic hydrocarbons (PAHs) are some of the most important pollutants (Figure 1). PAHs are hydrophobic semivolatile organic compounds and consist of two or more fused aromatic rings of carbon and hydrogen atoms, and the cycles can be oriented linearly, at an angle, or in clusters relative to each other [12]. Due to characteristic features such as their low solubility in water, high melting point, and resistance to nucleophilic attacks, PAHs are persistent pollutants and have a wide range of biological toxic effects [13,14]. In 2013, 16 PAHs were included in a list of high-priority environmental pollutants on the basis of their toxicity, potential to affect humans, and prevalence at contaminated sites [15,16,17].

**Figure 2:**
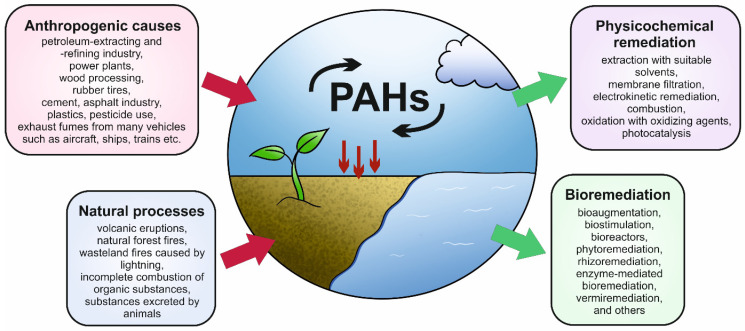
Main sources of PAHs and common methods of their remediation.

Although PAHs can enter the environment via natural processes (e.g., volcanic eruptions, forest fires, the incomplete combustion of organic substances, and as substances excreted by animals), increasing anthropogenic activity results in the substantial accumulation of these compounds in natural environments (Figure 2) [18,19,20]. PAHs enter the environment not only from the petroleum extraction and refinement industries but also from power plants, wood processing, and other numerous sources [5,11,19,20,21]. PAHs are equally present in aquatic and terrestrial ecosystems as well as in the atmosphere [22]. Most often, these chemical compounds are found in wastewaters and sewage-contaminated soils, thus having severe negative effects on clean freshwater reserves and agriculture [21]. Furthermore, soil systems tend to accumulate considerable amounts of PAHs because these substances have a high level of hydrophobicity (which increases with the number of condensed rings and with molecular weight) and are firmly adsorbed into soil particles [23,24].

Considering the wide range of sources and, as a consequence, the widespread occurrence of PAHs, researchers are increasingly focused on the problem of the effective and environmentally friendly remediation and restoration of sites contaminated with these substances. Many physical and chemical methods, such as UV oxidation, solvent extraction, and membrane filtration, are currently employed in practice for the cleansing of media contaminated with PAHs, but these techniques are usually expensive and require large chemical and/or energy inputs [25]. In contrast to these methods, approaches to the degradation of PAHs via biodegradation (bioremediation) are becoming a relevant and environmentally friendly option, whereby various microorganisms in the form of consortia, individual strains, or cell-free preparations of microbe-produced enzymes are used to reduce the concentration of pollutants [26,27,28].

Although most living organisms cannot survive in constant contact with high levels of PAHs, the populations of some microorganisms (bacteria, fungi, and algae) inhabiting chronically polluted sites possess several aerobic and anaerobic pathways for the degradation of aromatic and heterocyclic compounds, thus metabolizing them for assimilation as needed nutrients and energy sources [9,14,29,30,31,32]. To date, the biodegradation of PAHs by bacteria and fungi has been studied the most widely [33]. Numerous bacteria have been isolated and characterized in terms of the molecular mechanisms underlying their possible metabolic ability to degrade environmental pollutants [34,35]. At the same time, many investigators prefer to a search for the individual enzymes responsible for some stages of the degradation of complex mixtures of PAHs; this is because bioremediation technologies based on cell-free extracts containing enzymes or on immobilized/free enzymatic agents, including recombinant ones, have several advantages [36,37,38].

This review provides a brief description of sources of PAHs and evidence of their harmful effects on humans and on the environment. It should be noted that there are a huge number of PAHs; therefore, information on their sources and their individual toxicity as well as a discussion of the various types of remediation are shortly provided in Section 2, Section 3 and Section 4. The main part of the review is devoted to biological remediation (Section 5), including the application of individual microorganisms (Section 6.1), their consortia (Section 6.2), and cell-free enzymatic agents (Section 6.3). Due to the wide variety of aromatic compounds degraded by bacteria, their degradation typically is mediated by several enzymatic pathways leading to the formation of key intermediates. Therefore, classes of enzymes catalyzing the oxidation of PAHs are reviewed, and information on the study of these enzymes on individual model compounds such as phenanthrene, catechol, benzopyrene, etc., is presented. Most of the literary sources referenced in the writing of this review are scientific articles in peer-reviewed journals published during the 2014–2024 period.

## 2. Sources of PAHs

Although sources of PAHs in the environment can also be natural processes, anthropogenic sources still make a much greater contribution to overall pollution [12,39,40,41].

Incomplete combustion is the main source of PAH emissions in various industrial phenomena, such as waste incineration, rubber tire production, the asphalt industry, the production of some fungicides and insecticides, refinery exhaust gases, and power generation [12,27,39,40,41]. Other sources of industrial emissions include coal gasification and the use of electric arc furnaces, oxygen furnaces, diesel engines, and gasoline engines of large machinery [40,41]. Mobile sources of emissions include exhaust fumes from many vehicles, such as aircrafts, ships, trains, and all-terrain heavy and light automobiles [40,41]. Thus, the production of rubber tires, waste from oil refineries, electricity generation, coal gasification plants, automobile exhaust fumes, the burning of wood and coal, and agricultural waste represent a long but far from complete list of anthropogenic sources of PAH emissions [21].

In addition, being one of the most common structural units of organic compounds in nature, the benzene ring and its derivatives are widely employed in manufacturing, including the production of everyday products for the home, agricultural goods, and energy products. The electrochemical stability, durability, and hydrophobicity of PAHs make them ideal candidates for intermediates in thermoset plastics and lubricants [42]. PAHs are also used in finer technologies, such as the production of lithium-ion batteries [43].

According to their origin, sources of PAHs are also classified into pyrogenic, petrogenic, and biogenic [39]. Pyrogenic PAHs are generated by the unintentional incomplete combustion of organic materials and by pyrolytic processes aimed at the thermal breakdown of complicated petroleum-related compounds into lighter hydrocarbons. Petrogenic PAHs are present in petroleum and its byproducts, which are widespread due to the storage, transportation, applications, and leakage of crude oil and refinery products [12]. Some living organisms, such as algae and plants, and some microorganisms are also capable of producing PAHs, which, in this case, are categorized as biogenic [44].

## 3. Toxicity of PAHs

PAHs have been found to have carcinogenic, immunotoxic, cardiotoxic, and mutagenic properties [45,46,47]. These chemical compounds have adverse effects not only on human health but also on the environment [5,48,49]. PAHs have an ecotoxic impact on aquatic flora and fauna and on birds [21]. PAHs released into water bodies are known to cause severe developmental disorders in fish embryos, thereby causing damage to aquatic ecosystems and the fishing industry [50].

Epidemiological studies have linked long-term exposure to PAHs with various adverse health effects, such as diabetes mellitus, cardiovascular diseases, and cancers [51,52,53]. The toxicity of PAHs to living organisms, in particular mammals, is largely due to the formation of reactive intermediates during the catalytic oxidation of these compounds in the liver by cytochrome P450 and other oxidases. The resultant PAH oxidative metabolites, such as diol epoxides, cationic radicals, hydroxyalkyl derivatives, and *o*-quinones, have high levels of electrophilic reactivity. Thus, these metabolites can form covalent adducts with DNA, leading to the emergence of mutations in DNA and changing gene expression profiles and ultimately inducing the malignant transformation of cells [12,54].

Due to their hydrophobic and adsorptive properties, PAHs from the atmosphere and from water sources enter soil and accumulate there; from contaminated soil, these chemical compounds are transferred into groundwater, plants, and food [21]. For many people, their primary exposure to PAHs occurs in the workplace, e.g., in industrial settings or through the regular inhalation of vehicle exhaust fumes and road dust containing PAHs [13]. Moreover, cigarette smoking, the drinking of contaminated water, and the consumption of fried and smoked foods may be risk factors too [13,21].

## 4. Physicochemical Methods of Remediation

Present-day approaches to the remediation of PAHs and other hydrocarbons that are components of petroleum-related pollution can be subdivided into four main categories: chemical, thermal, physical, and biological remediation [5].

### 4.1. Physical Approaches

One of the options for the physical removal of PAHs from water, sludge, or soil is extraction with suitable solvents such as acetone, alcohol, hexane, dichloromethane, methyl ethyl ketone, or toluene [21]. Nontoxic and biodegradable extraction agents such as cyclodextrins, vegetable oils, and humic acid can also be employed for soil washing [25]. Additionally, lately, there has been a discussion of soil washing with extraction agents in combination with other techniques, including the use of surfactants to change the solubility of PAHs [55]. It is worth pointing out that the effectiveness of soil washing with a surfactant strongly depends on the properties of the PAHs, the structure of the surfactant, and the soil composition [25,55].

Various membrane filtration methods, such as ultrafiltration, micro/nanofiltration, and reverse osmosis, can be employed to remove PAHs from water [21,56,57,58]. Furthermore, some adsorbents, such as activated charcoal, biochar, magnetic nanomaterials, and graphene oxide, are used quite successfully to remove PAHs from water and soil [59,60]. When adsorbents are employed, the efficiency and rate of removal of PAHs are highly dependent on such factors as the reaction temperature, pH, humidity, and adsorbent concentration [21].

An interesting physical approach to PAH removal/degradation is electrokinetic remediation. This technique can be used with in situ PAH-contaminated soils that have lower levels of hydraulic conductivity through the application of a direct low-intensity electrical current via electrodes. The efficiency of electrokinetic remediation is significantly improved by the simultaneous use of solvents, surfactants, and vegetable oils. This method also becomes more cost-effective when applied together with in situ biodegradation techniques [23].

### 4.2. Thermal Approaches

Thermal technologies involve the combustion of PAHs from contaminated soil or from industrial waste at high temperatures (900–1200 °C); as a result, the PAHs degrade or are transferred into a gas phase and are captured in special traps [23,25]. The main disadvantage of combustion technology is its high energy consumption, which makes it costly.

### 4.3. Chemical Approaches

Another popular approach to PAH removal/degradation is chemical remediation. One of the most commonly used options is PAH oxidation with oxidizing agents such as ozone, Fenton’s reagent (Fe^2+^ + H_2_O_2_), potassium permanganate, a peroxyacid (R-COOOH), hydrogen peroxide, or activated sodium persulfate [21]. All these oxidizing agents have some disadvantages. The application of Fenton’s reagent requires a substantially acidic pH (2.8–3.0) during the oxidation process and during the removal of Fe^3+^ after the oxidation; therefore, this method is impractical, expensive, and detrimental to soil quality and to the physiological processes of endemic microorganisms [39]. Modified Fenton oxidation at a neutral pH by means of chelating agents can partially mitigate these limitations [21]. The efficiency of the oxidation of PAHs by ozone strongly depends on the water content of soil; in addition, high-molecular-weight PAHs are resistant to oxidation by ozone and by Fenton’s reagent [25].

The oxidation process is influenced by various factors such as temperature, pH, the choice of oxidant, and soil type [15]. A shortage of an oxidizing agent during the treatment of PAH-contaminated soils gives rise to intermediate products whose further oxidation is difficult, and this situation in itself becomes a source of new pollution [21].

In recent years, the attention of researchers has increasingly been focused on the development of approaches to a so-called improved oxidation process. During this process, agents that produce radicals such as hydroxyl (OH·) or sulfate (${SO}_{4}^{-}\cdot )$ are activated. For example, persulfate and peroxymonosulfate can be activated by heat, alkali, UV light, activated charcoal, transition metals (such as Fe, Cu, and Co), ultrasound, and hydrogen peroxide [61]. Radical-driven oxidation enables the degradation of pollutants without the formation of toxic intermediates, while carbon dioxide and water are generated as oxidation products [62].

Photocatalysis is also an important approach to the removal of PAHs from the environment. Among effective photocatalytic materials for the removal of PAHs are titanium dioxide and zinc oxide [63,64]. In addition, photocatalysis with the help of semiconductor quantum dots based on a hybrid of indium phosphide and zinc sulfide has shown a high efficiency in the degradation of PAHs such as phenanthrene, naphthalene, and pyrene [65].

Despite the appreciable effectiveness of physical methods of PAH remediation, eventually, all these techniques simply lead to the extraction of these chemical compounds from water and soil but do not convert the pollutants into less hazardous ones; hence, these methods remain suboptimal. Chemical remediation—though offering the targeted removal of contaminants—leaves behind a series of toxic byproducts [66]. Additionally, many physical and chemical methods are usually costly and energy-intensive [25].

## 5. Bioremediation

Owing to the substantial disadvantages of physicochemical methods for PAH removal/degradation, e.g., the cost and the unattainability of complete degradation, bioremediation approaches have been attracting more attention from researchers lately [19,67]. Bioremediation has several advantages because it is an environmentally friendly process that is also cost-effective and promotes the sustainable restoration of an ecosystem and therefore represents an effective way to remove PAHs from contaminated aquatic environments and from soils [23,68,69]. Bioremediation methodologies are characterized by their low technological costs and allow for the complete mineralization of xenobiotics. Due to the efficient degradation of PAHs by microorganisms, pollutants are transformed into less toxic compounds, aside from water and CO_2_ [70]. Bioremediation does not have a considerable impact on a natural environment and is effective at removing pollutants even at low concentrations [71,72].

In general, bioremediation methods can be categorized into ex situ and in situ ones. Ex situ techniques require the physical transfer of contaminated materials to another site for subsequent processing, whereas in situ methods involve the processing of a contaminant in situ and are preferred due to their lower costs and ability to remove contaminants on a regular basis [21].

The bioremediation of PAHs by means of microorganisms (bacteria, fungi, or algae), and sometimes plants includes a number of approaches. Among them, the popularity of bioaugmentation and biostimulation is the highest, followed by the use of bioreactors, phytoremediation/rhizoremediation, enzyme-mediated bioremediation, vermiremediation, and others [23]. The distinction between some of the above approaches is quite arbitrary because they are often utilized in combination, or, as in the case of bioreactors, they represent a special case of bioaugmentation.

Biostimulation is an enhancement of the PAH biodegradation activity of local microorganisms via supplementation with nutrients (sources of N, P, S, and K), fertilizers, organic waste, or other substances. This approach is mainly applied to overcome limitations on the active growth of microorganisms capable of degrading PAHs [21]. Biostimulation can also be implemented through the adaption of microorganisms to pollutants. To this end, endemic crops are enriched with high concentrations of target pollutants for adaptation and the directed selection of more efficient microbial strains [73].

Phytoremediation is an approach that is based on plants rather than microorganisms for the removal of PAHs from soils. The use of plants is feasible because they can eliminate organic pollutants via such mechanisms as phytoextraction (the removal of pollutants from soil), phytovolatilization (an atmospheric release of volatile pollutants (absorbed from soil) through plant organs), and phytodegradation (the degradation of pollutants by enzymes secreted by a plant and/or microbes associated with the plant) [74,75]. Moreover, plants promote soil aeration, increasing soil permeability, which can increase the aerobic biodegradation of PAHs [21].

Rhizoremediation is some combination of phytoremediation and bioaugmentation, because under this scenario, rhizosphere microorganisms associated with plants are employed to degrade PAHs [21,76]. During rhizoremediation, plant roots supply nutrients for the growth and activity of the microbes that degrade PAHs. In the meantime, microorganisms ensure plant growth on an unfavorable substrate by reducing its phytotoxicity in the root zone [21].

Among the advantages of phyto- and rhizoremediation of PAHs are the preservation of a natural state of soil, their low energy consumption owing to the efficient utilization of sunlight, and the achievement of large volumes of microbial biomass in the soil. The main disadvantages of these approaches are the limited depth of restoration, their applicability only to lightly contaminated sites, the problem with recycling PAHs accumulated in plant organs, and the risk of pollutants getting into the food chain [25,55].

Another interesting approach to PAH bioremediation is vermiremediation. Earthworms can remove these chemical compounds from soil through skin absorption or intestinal digestion, during which the pollutants are transformed or degraded into harmless compounds [77,78]. Vermiremediation improves the physical/biological characteristics of soil and promotes the proliferation of beneficial soil microorganisms [79]. On the other hand, a serious limitation of this methodology is the ability of earthworms to survive only at relatively low concentrations of pollutants [23].

Bioaugmentation involves the introduction of an inoculum of an individual microorganism or a set of microbes that degrade pollutants to achieve optimal degradation and sometimes to improve the catabolic capabilities of indigenous microbes [21]. For bioaugmentation, individual microorganisms (bacterium, fungus, or alga) or various combinations thereof are used. In this regard, the degradation of PAHs with the help of bacteria and fungi has been investigated widely.

The bioremediation of PAHs by means of fungi is being actively researched, similar to bacterial bioremediation [80,81]. Apparently, unlike bacteria, fungi do not utilize PAHs as the only carbon source during degradation. Rather, the cometabolism of these chemical compounds proceeds with the formation of various oxidized products, including CO_2_. The key enzyme for PAH degradation in fungi is monooxygenase. In general, two main kinds of fungi used for PAH bioremediation have been described, namely, lignolytic fungi (white rot fungi) and nonlignolytic fungi [21]. Lignolytic fungi possess a few enzymes specialized for lignin degradation, including lignin peroxidases, manganese peroxidases, and laccases, which allow them to oxidize PAHs to biphenolic intermediates that are ultimately oxidized to quinones [82]. By contrast, nonlignolytic fungi produce enzymes similar to cytochrome P450 monooxygenase that are capable of oxidizing PAHs [19]. Nevertheless, the direct application of fungi for the in situ bioremediation of PAHs has several limitations, such as insufficient biomass growth and the lack of established methodologies [21].

## 6. Bacterial Bioremediation of PAHs

### 6.1. Individual Bacterial Strains

Evans et al. were the first to demonstrate the bacterial degradation of phenanthrene by *Pseudomonas aeruginosa* via the “ring fission” mechanism [83]. Since then, genes responsible for PAH degradation have been studied in a wide range of other Gram-negative and some Gram-positive bacterial strains. Some of the strains, mentioned in the current review, are listed in Table 1. Bacteria belonging to the genera *Arthrobacter*, *Stenotrophomonas*, *Acidovorax*, *Rhodococcus*, *Ochrobactrum*, *Microbacterium*, *Brevibacterium*, and *Burkholderia* are reported to be able to degrade phenanthrene [9,84]. Lily et al. have found that *Bacillus subtilis* BMT4i has an ability to degrade various PAHs such as naphthalene, anthracene, dibenzothiophene, and high-molecular-weight benzo[*a*]pyrene molecules [85]. The complete degradation of anthracene by bacteria from the genera *Beijerinckia*, *Mycobacterium*, *Nocardia*, *Pseudomonas*, *Sphingomonas*, and *Rhodococcus* has been documented, with the initial intermediate being an oxygenated dihydriol [86]. Some articles have addressed the activity of strains of the actinobacterium *Rhodococcus opacus* in relation to the degradation of phenol and of some intermediate compounds arising during this process, in particular, catechol [87,88]. Muneeswari et al. recently reported a petrophilic strain of *Enterobacter xiangfangensis* STP-3 that can degrade up to 82% of PAHs in just 14 days [89]. Wang et al. have found strains of *Bacillus amyloliquefaciens*, *Citrobacter* sp., and *Brucella melitensis* to effectively degrade petroleum hydrocarbons, methylphenanthrenes, methyltriaromatic steroids, triaromatic steroids, dimethylphenanthrenes, trimethylphenanthrenes, dimethyldibenzothiophenes, and methyldibenzothiophenes [90]. From samples of seawater contaminated with crude oil, Lazzem et al. have isolated a bacterium *Achromobacter aegrifaciens* strain that is capable of effectively decomposing a high-molecular-weight PAH called chrysene as a sole source of carbon and energy [91].

To date, the number of genera featuring the identified bacteria that can degrade various components of petroleum hydrocarbons is around 80 [66]. An analysis of the data on the activity of various strains in terms of PAH degradation has shown that many of the isolates that decompose polluting compounds of this type are bacteria belonging to the genera *Pseudomonas* (Gammaproteobacteria) and *Sphingomonas* (Alphaproteobacteria). Isolates affiliated with these genera carry numerous genes, operons, and large plasmids that enhance metabolic diversity and catabolic versatility, thereby promoting the degradation of diverse aromatic compounds [108]. Some bacteria also synthesize secondary metabolites, namely biosurfactants, which make petroleum-derived pollutants more bioavailable [109]. Biosurfactants reduce surface tension at the oil–water interface and allow difficult-to-degrade components, such as PAHs, to more easily enter the aqueous phase, where they can then be metabolized by bacteria [110].

Water and soil samples collected at sites affected by long-term and/or regular industrial pollution are becoming the key sources of microbes that can effectively decompose PAHs; the reason for this is that living under such conditions stimulates the directed evolution of enzymatic systems that lead to PAH catabolism in bacteria. In this context, scientists are constantly searching for new sources of more effective, versatile, and resistant strains, thus expanding the range of study site types. Cauduro et al. have identified four strains that belong to the *Burkholderia cepacia* complex and efficiently degrade benzo[*a*]pyrene in activated-sludge samples from a petrochemical wastewater treatment plant [97]. Their study revealed the good potential of activated sludge from wastewater treatment plants as a source of bacterial consortia and individual isolates for PAH bioremediation. Tidal shallows near Sinduri Beach in Taean, South Korea, which were damaged by an oil spill in 2007, have provided new bacterial strains (from *Cobetia marina*, *Rhodococcus soli*, and *Pseudoalteromonas agarivorans*) that can degrade PAHs [98].

Sarkar et al. have stated that the *Pseudomonas* and *Bacillus* spp. strains that are found initially in samples collected near oil refineries are replaced—after subsequent enrichment with oil waste—by bacteria from the genera *Burkholderia*, *Enterobacter*, *Kocuria*, *Pandoraea*, and others. Strains isolated after prolonged enrichment of such samples demonstrate a high efficiency in the degradation of aliphatic and polycyclic hydrocarbons as well as a greater production of biosurfactants [111]. On the other hand, data from other investigators indicate the predominance of *Bacillus* spp. when samples are enriched with various PAHs. Abdelhaleem et al.—by applying the enrichment method to samples collected from an industrial area in Egypt—have isolated four Gram-positive bacterial strains (from *Bacillus anthracis*, *B. cereus*, *B. mojavensis*, and *B. subtilis*) that effectively degrade anthracene, α-naphthol, and catechol [95]. In their work, the strain *B. subtilis* EMCC 4454 manifested the greatest efficiency in the degradation of mutagenic and carcinogenic anthracene. An individual catechol-1,2-dioxygenase of *B. subtilis* also showed a high efficiency of anthracene oxidation. In their experiments, the expression level of catechol-1,2-dioxygenase rose 23.2-fold during 72 h of incubation with PAHs [95]. On the other hand, in a study by Benedek et al., when samples were enriched with naphthalene in the presence of (and at reduced concentrations of) oxygen, *Pseudomonas* spp. became predominant representatives of the bacterial community [112].

An assay of samples collected from a mud volcano in Moldova indicates that mud volcanoes can be natural sites of PAH emissions and may serve as a source of microorganisms capable of degrading PAHs. Namely, Remizovschi et al. noticed that in the PAH-saturated sediments of a mud volcano, there are many strains related to known bacterial taxa that decompose PAHs, e.g., Enterobacteriaceae, Methylobacteriaceae, Bradyrhizobiaceae, Oxalobacteraceae, Comamonadaceae, and Sphingomonadaceae [113].

Areas with extreme marine conditions are potential sources of new microbial isolates featuring dynamic metabolic activity. *Dietzia psychralcaliphila* J1ID has been isolated from sediments from Deception Island, Antarctica, that have been grown on phenanthrene [9]. In the paper just cited, within liquid media, *Dietzia psychralcaliphila* J1ID showed an 84.66% degradation of phenanthrene. Whole-genome sequencing uncovered the presence of a wide range of monooxygenase and dioxygenase genes as well as catabolic dehydrogenase genes, which collectively provide the genomic basis for the biodegradation of polyaromatic pollutants [9].

It is worth highlighting the strategy of searching for (and exploiting) bacteria originating from a site where PAH bioremediation is necessary. On the one hand, these bacterial strains already have mechanisms of adaptation to specific environmental conditions. On the other hand, the use of endemic microorganisms is sometimes mandatory due to the prohibition of introduction of alien organisms into special-status areas such as Antarctica [114,115,116,117]. In 2017, Gran-Scheuch et al. isolated several bacteria metabolizing PAHs from diesel-contaminated Antarctic soil samples. The isolate *Sphingobium xenophagum* D43FB showed a high efficiency in phenanthrene degradation. Furthermore, D43FB was able to degrade phenanthrene in the presence of its common copollutant, the heavy metal cadmium. Thus, the analysis of samples collected at naturally contaminated sites revealed a strain that has promising potential for the bioremediation of diesel-contaminated Antarctic ecosystems [107].

The main limiting factor for the application of bioremediation is a difficulty with optimization owing to an appreciable influence of environmental factors [118]. Therefore, one of important areas of PAH bioremediation is the use of bacterial extremophiles, such as halophilic, acidophilic, or thermophilic bacteria. An elevated temperature enhances the diffusion of PAHs because of a decrease in viscosity, thereby ultimately increasing the bioavailability of these chemical compounds; hence, the use of thermophiles is rather relevant [69]. In the work just cited, researchers isolated the thermophilic strains *Aeribacillus pallidus* UCPS2, *Bacillus axarquiensis* UCPD1, *Bacillus siamensis* GHP76, and *Bacillus subtilis* subsp. *inaquosorum* U277 from samples collected at the Unkeshwar hot springs (India); these strains manifested a high efficiency in the degradation of a PAH mixture (anthracene, fluorene, phenanthrene, and pyrene) at 50 °C within a consortium [69].

Ecological niches with extreme conditions, such as saline soils, are most vulnerable to petroleum pollution owing to their close association with the oil industry [119]. The bioremediation of PAHs under such conditions requires microorganisms resistant to extreme conditions. The strain *Pelagerythrobacter* sp. N7, which has been isolated from soil samples contaminated with PAHs and containing elevated salt concentrations, shows a high efficiency in PAH degradation across a wide salinity range (1–10%) [103].

In recent years, a few articles have come out about the suitability of archaea for PAH degradation [120]. To date, the degradation pathways and mechanisms underlying bioremediation by archaea have not been studied widely [121]. The paper just cited presents the discovery of the halophilic archaeon *Natrialba* sp. C21, which is capable of the degradation of phenol, naphthalene, and pyrene at elevated salt (NaCl) concentrations.

The pollution of industrial areas with PAHs is often combined with the presence of other polluting compounds, including heavy metals. Heavy metal pollution itself can negatively affect the bioremediation of hydrocarbon compounds owing to the synergistic cytotoxic effect of the two types of pollutants [122,123]. Consequently, it is becoming topical and relevant to search for bacterial strains capable of decomposing PAHs while having a high resistance to the presence of heavy metals in soil. In soil contaminated with petroleum products, Kotoky et al. have discovered the bacterial strain *Serratia marcescens* S2I7, which, along with the ability to decompose benzo[*a*]pyrene, is also tolerant to the presence of cadmium [106]. The strain *Bacillus subtilis* SR1, which is capable of the degradation of PAHs and resistant to several heavy metals, has been isolated from rhizosphere soil contaminated with petroleum [96]. Moreover, among the rhizobacteria that stimulate plant growth, the strain *Pseudarthrobacter* sp. L1SW has been found to have a high phenanthrene degradation efficiency and is resistant to the presence of heavy metals (Ni, Zn, and Cr); it is also noteworthy that the strain L1SW degrades phenanthrene through a unique pathway, generating intermediate metabolites including *cis*-3,4-dihydrophenanthrene-3,4-diol, 1-hydroxy-2-naphthoic acid, and phthalic acid [104]. The *Cupriavidus* sp. MTS-7 strain has been isolated from soil samples from a site of a former gas plant (in Australia), contaminated for a long time with PAHs and heavy metals, and has been proposed as a promising candidate for the bioremediation of mixed types of contamination with PAHs. In that work, MTS-7 demonstrated a very high degree of degradation of benzo[*a*]pyrene (as compared to other known bacteria that mineralize PAHs) and an ability to degrade PAHs across a wide pH range, especially acidic pH levels, and in the presence of low concentrations of Cu, Pb, Zn, and Cd [99].

Overall, the available evidence indicates a relatively low tolerance to the presence of heavy metals in such orders as Sphingobacteriales, whereas Xanthomonadales and Burkholderiales demonstrate a greater resistance to heavy metal pollution. In this context, the introduction of resistant strains into a bacterial consortium can improve the total tolerance of the consortium, e.g., owing to the high efficiency of the horizontal transfer of genes responsible for resistance to heavy metals [118].

Although most bacterial consortia respond to the presence of heavy metals with a general decrease in metabolic activity and in metapopulation diversity, at the same time, an elevated rate of PAH decomposition is observed in the presence of heavy metals. The mechanism of this effect has not yet been identified, but it is believed that in the presence of heavy metals, there is an enhancement of the activity of enzymes that are crucial for the biological degradation of PAHs: dioxygenases [118,124].

A possible cost-effective bioremediation strategy is the isolation of new bacterial strains from contaminated soils that not only exhibit broad substrate specificity in terms of PAH degradation but also have a high tolerance to heavy metals, extreme environmental conditions, and severe deficits of nutrient sources.

Another topical problem—that must be taken into account when an effective strategy is being developed for the bacterial remediation of PAHs—is the presence of a multicomponent mixture of these chemical compounds, which is common in contaminated environments. Given that the activity of certain strains is often assayed using separate substrates that model contamination with PAHs, due attention must be given to the possibility of the degradation of several PAHs at once. Aside from the fact that PAH biodegradation becomes more difficult with increasing complexity of these molecules, in a complex mixture of such compounds, chemical interactions between its components also take place. Sakshi et al. have demonstrated the effectiveness of *Kocuria flava* and *Rhodococcus pyridinivorans* strains in degrading a mixture of PAHs (phenanthrene, anthracene, fluorene, and pyrene) [28]. Additionally, during the degradation of the multicomponent mixture, their study showed an overexpression of the genes responsible for PAH catabolism. Furthermore, the expression of various enzymes differed between the two strains, pointing to the good potential of their joint use for the bioremediation of a mixture of PAHs [28].

Ren et al. have investigated the activity of the strain *Arthrobacter* sp. YC-RL1 against various aromatic compounds [93]. The strain YC-RL1 effectively degraded *p*-xylene, naphthalene, phenanthrene, biphenyl, *p*-nitrophenol, and bisphenol A alone or in a mixture. Moreover, YC-RL1’s genome was analyzed for the genes responsible for certain branches of the pathway for the metabolism of aromatic compounds; an enzyme called 2,3-dihydroxybiphenyl-1,2-dioxygenase was isolated, and its activity was confirmed by heterologous expression.

One of the options for using bacteria for PAH bioremediation is the immobilization of the microbes. This approach ensures the stability of the strains employed, protection from suboptimal environmental factors, and reduced competition with indigenous bacteria [125,126]. For instance, the immobilization of *Pseudomonas taiwanensis* PYR1 and *Acinetobacter baumannii* INP1 on slag balls leads to the higher degradation of pyrene and its derivatives in soil contaminated with petroleum [92]. The highest degradation of PAHs by a culture of the bacterium *Rhodococcus rhodochrous* ATCC 21198 has been achieved with immobilized cells in the presence of a surfactant: Tween 80 [105]. Emelyanova and Solyanikova have tested a biosensor based on immobilized cells for assessing the phenol hydroxylase activity and the activity of phenol-transporting cellular systems of *Rhodococcus opacus* 1CP [87].

The properties of some discovered strains make it possible to use them not only to clean soils contaminated with PAHs but also for other purposes. For instance, Rajkumari et al., having analyzed the genome of the strain *Klebsiella pneumoniae* AWD5, noticed that this strain possesses not only several genes responsible for the degradation of PAHs (and of some other xenobiotics) but also genes that can promote the growth of plants [101,102]. These hypotheses were proven experimentally, and *K. pneumoniae* AWD5 was found to enhance the growth of *Jatropha curcas* in the presence of soil contaminated with pyrene [101,102].

### 6.2. Consortia of Microorganisms

Much evidence indicates that a microbial consortium is an effective tool for the bioremediation of PAH-contaminated soils [127,128]. Tripathi et al. have demonstrated a higher efficiency of the degradation of three-, four-, and five-ring PAHs by a bacterial consortium as compared to a stand-alone strain of *Xanthomonas boreopolis*, *Microbacterium schleiferi*, *Pseudomonas aeruginosa*, or *Bacillus velezensis* [66]. Kumari et al. have reported strains of *Stenotrophomonas maltophilia*, *Ochrobactrum anthropi*, *Pseudomonas mendocina*, *Microbacterium esteraromaticum*, and *Pseudomonas aeruginosa* that can degrade several PAHs [84]. Of note, in their experiments, the degradation efficiency of the consortium of bacterial isolates was significantly higher than that of individual bacterial isolates. Similarly, Kotoky and Pandey have registered better degradation by a consortium than by individual isolates, possibly owing to a synergistic effect of multiple isolates working together to degrade PAHs [129].

Vaidya et al. have designed a bacterial consortium that effectively decomposes such high-molecular-weight and biodegradation-resistant PAHs as chrysene. Their consortium, consisting of the bacterial strains *Rhodococcus* sp. ASDC1, *Bacillus* sp. ASDC2, and *Burkholderia* sp. ASDC3, when chrysene served as the sole carbon source, achieved a maximum degradation rate of PAHs up to 1.5 mg/L per day [94]. Patel et al. have devised a mixed bacterial culture of 22 bacterial genera (among which representatives of *Azoarcus* and *Chelativorans* predominated) that is capable of decomposing phenanthrene and fluoranthene in an aqueous solution with a concentration of PAHs up to 400 mg/L [130]. A whole-genome sequencing analysis of two new bacterial strains *Klebsiella michiganensis* EF4 and *K. oxytoca* ETN19 allowed Lou et al. to determine that both strains uniquely degrade phenanthrene through a putative pathway that catabolizes 2-carboxybenzalpyruvate and feeds the products into the tricarboxylic acid cycle. In their work, a combination of the two strains significantly increased the efficiency of phenanthrene degradation as compared to the stand-alone strains [100]. Książek-Trela et al. have investigated the degradative potential of several commercially available bacterial consortia to bioremediate a mixture of the 13 PAHs most commonly found in soils; the greatest efficiency of degradation was achieved for fluorene when a mixture containing eight strains of bacteria from the genus *Bacillus* was used: *B. coagulans*, *B. amyloliquefaciens*, *B. laterosporus*, *B. licheniformis*, *B. mucilaginosus*, *B. megaterium*, *B. polymyxa*, and *B. pumilus* [131].

Individual strains capable of degrading PAHs possess enzymes specialized for the oxidation of individual substances; however, the enhanced degradation achieved by means of a consortium can be ascribed to cometabolic activity. Cometabolism involves the transformation of hydrocarbons as a secondary substrate while the primary substrate is metabolized. Synergistic effects in a consortium may result from an interaction of different strains which promotes the more efficient and diverse enzymatic degradation of polyaromatic hydrocarbons [66]. Additionally, the use of mixed cultures, which have a wider range of metabolic properties, is recommended to prevent the formation of toxic intermediates during PAH bioremediation [132]. In the degradation of PAHs by the strain *Rhodococcus rhodochrous* ATCC 21198, the formation of various oxidation products has been detected, whose toxicity has turned out to be higher than that of the starting compounds [105]. Consequently, the application of this strain to bioremediation will be more appropriate within a consortium, where other participants will be able to process the toxic metabolites released by *Rhodococcus rhodochrous*.

### 6.3. Enzymatic Degradation

Enzymatic bioremediation involves the use of isolated enzymes from bacteria, fungi, and other living organisms to remove PAHs. An isolated enzyme is extremely efficient and selective due to a higher reaction rate and the ability to catalyze reactions across a wide range of temperatures and pH levels. Furthermore, the advantages of the enzymatic bioremediation of PAHs include a narrow substrate specificity (which can lead to the faster degradation of certain contaminants) and the greater independence of the enzymatic reaction from supplementary nutrients added to the reaction mixture [70]. These factors are important because a contaminated environment suitable for bioremediation strategies may contain—aside from a mixture of various PAHs—various organic salts and heavy metal ions (usually oxidized by combustion) and usually has a neutral or acidic pH level [3,18]. This state of affairs in turn imposes certain requirements for the properties of the enzymes employed for bioremediation: an elevated stability, broad substrate specificity, and high catalytic efficiency [37,133].

The microbial degradation of such hydrophobic compounds as PAHs is possible due to the presence of key metabolic enzymes that function in a sequential manner and as a cohort [19]. The mechanisms developed by microorganisms for the assimilation of aromatic compounds have been fixed and optimized by natural selection, causing the emergence of specific corresponding enzymes and their arrangement within functional enzymatic cascades. Due to the wide variety of aromatic compounds degraded by bacteria, this degradation typically is mediated by several peripheral pathways leading to several key intermediates, which are then incorporated into cellular metabolism by the conversion to cellular housekeeping metabolites such as acetyl coenzyme A (CoA), succinyl-CoA, or pyruvate [31].

Given that aromatic compounds—owing to the delocalization of their π orbitals—are highly stable, key degradation steps include the activation of the aromatic ring through the introduction of substituents and dearomatization. In aerobic microorganisms, the activation of an aromatic substrate is usually implemented by hydroxylation reactions, and the critical dearomatization step is carried out by dioxygenases that cleave the ring. It is ring-cleaving dioxygenases that are thought to catalyze key reactions in the aerobic microbial degradation of aromatic compounds. There are currently three main classes of known ring-cleaving dioxygenases. Dioxygenases that perform the intradiol- or extradiol-type cleavage of the aromatic ring of catechol and of its derivatives belong to classes I and II, respectively. The oxidation reaction catalyzed by these enzymes produces either *cis*,*cis*-muconic acid and its derivatives (class I, *ortho* cleavage) or 2-hydroxymuconate semialdehyde derivatives in the case of *meta* cleavage (class II) [134,135,136]. Class III ring-cleaving oxygenases require two hydroxyl groups in a *para* orientation, as in gentisate and its derivatives [137,138]. The products of gentisate oxidation by third-class dioxygenases or gentisate dioxygenases are 3-maleylpyruvate and its derivatives [139]. The different classes of ring-cleaving dioxygenases seem to have evolved relatively independently because there is no significant sequence homology between the individual classes [140,141]. Nonetheless, class II (extradiol) and class III dioxygenases share some similarities. For example, both classes contain catalytically active Fe(II) ions in their active sites. By contrast, class I intradiol dioxygenases contain Fe(III) ions in their active sites [135].

Thus, during the stages of PAH degradation, a variety of catechol derivatives or structures are usually formed, including catechol-like fragments as well as gentisates, which undergo cleavage at certain positions depending on the class of dioxygenases. These dioxygenases play an important role in the degradation of aromatic compounds by soil bacteria. These enzymes may even be key determinants of the fate of some aromatic compounds in the environment because their catalytic activity determines the effective decomposition of complicated PAHs to nontoxic compounds that can become involved in the tricarboxylic acid cycle [139].

Bioinformatic analysis is an important modern tool for the search for new strains of bacteria and enzymes that can be effectively employed in bioremediation. The investigation into possible pathways of the biodegradation of various pollutants has been an urgent task for a long time. Nonetheless, there are still many unsolved problems in this area and a wide field for further research on the creation and use of such enzymes, even though many papers have already been published describing certain microorganisms [9,83,86] or metabolic pathways of the degradation of pollutants in such organisms [107,142,143,144] as well as individual enzymes processing the main intermediates of PAH biodegradation [87,145,146].

The search for new genomic sequences and new enzymes enables scientists not only to discover enzymes with unique properties but also to examine the diversity of these enzymes and to utilize this information to obtain stabler and more effective enzyme preparations with a broad substrate specificity. For example, salicylate-1,2-dioxygenase from *Pseudaminobacter salicylatoxidans* BN12 (psSDO) is a class III ring-cleaving dioxygenase belonging to the cupin superfamily, along with other gentisate-1,2-dioxygenases or 1-hydroxy-2-naphthoate dioxygenases [137,147,148,149,150]. However, psSDO markedly differs from other enzymes in its class owing to a unique ability to oxidatively degrade salicylate, gentisate, and 1-hydroxy-2-naphthoate [149]. A crystallographic structural analysis of complexes of psSDO with various substrates has helped to identify the amino acid residues that are responsible for the broad substrate specificity of the enzyme [149]. The same authors also reported that a single amino acid substitution (Gly106Ala) in psSDO within the region of the enzyme active center “narrows” its substrate specificity to that of known “regular” gentisate-1,2-dioxygenases [149], and this finding opens up opportunities for “broadening” the specificity of related enzymes.

Wang et al. have created several genetically engineered versions of biphenyl dioxygenase from *Burkholderia xenovorans* LB400; these enzymes possess an enhanced activity toward dibenzofuran as compared to the wild-type enzyme [151]. It was found in that work that the substitution of Ser283Met in the biphenyl dioxygenase affects the regiospecificity of the formation of a dibenzofuran oxidation product. A structural analysis indicated that amino acid residue Met283 is critical for increasing the flexibility of the catalytic pocket and for the attainment of an optimal orientation of dibenzofuran for an efficient catalytic reaction [151].

Nagy et al. have conducted a large-scale bioinformatic study to search for new potential enzymes for PAH biodegradation using metagenomic data deposited in the NCBI SRA archive by means of soil samples contaminated with various types of industrial waste from coal gasification plants [33,152]. Due to the bioinformatic methods, these authors were able to identify genes of new potential oxygenases that can oxidize various PAHs. In particular, two dioxygenases (PAH1_99 and PAH1_105) and one catalase-peroxidase (PAH6_39) were isolated and characterized, and their ability to degrade various PAHs was evaluated. This work exemplifies how bioinformatic and biochemical approaches can be combined in a directed search for enzymes with specific properties.

Benzo[*a*]pyrene, being a typical representative of PAHs, poses a serious danger to human health and to the environment. Nonetheless, there are still very few data in the literature on the pathway of the bacterial degradation of benzo[*a*]pyrene. Qian et al. have carried out extensive work and found that the strain *Pontibacillus chungwhensis* HN14—isolated from a high-salt environment and demonstrating a strong ability to degrade benzo[*a*]pyrene—converts this compound into chrysene, phenanthrene, and naphthalene. In this case, monooxygenase CYP102(HN14) plays a key part in the initial oxidation of benzo[*a*]pyrene, and a further conversion takes place due to epoxide hydrolase EH(HN14) [153]. This very recent publication underscores that there are still many knowledge gaps in the field of the bacterial remediation of PAHs, and the missing pieces of the puzzle require careful and comprehensive research.

One of the options for using enzymatic preparations for the cleanup of PAH-contaminated environments is immobilized enzymes. For instance, Silva et al. have studied the activity of catechol-1,2-dioxygenase from *Mycobacterium fortuitum* under various reaction conditions [154]. These authors found that the immobilization of the enzyme on calcium alginate helps to maintain its activity within wider pH ranges and in the presence of certain ions that inhibit the enzymatic activity in a cell-free extract. Moreover, the duration of the enzymatic activity of catechol-1,2-dioxygenase and the temperature optimum for its functioning increased after the immobilization. Extreme conditions, such as a suboptimal environmental pH and the presence of heavy metals, can occur in contaminated types of waste, suggesting that immobilization on a cheap, nontoxic support or by simple manipulations such as calcium alginate can turn this enzyme into an effective biotechnological alternative for the processing of liquid waste contaminated with aromatic compounds [154].

An enzyme called chlorocatechin-1,2-dioxygenase from *Pseudomonas putida* is of particular interest because it is capable of oxidizing a wider range of substrates than other dioxygenases, including halogenated compounds such as 3-chlorocatechol, 4-chlorocatechin, 4-fluorocatechin, 3,5-dichlorocatechin, 3,5-dibromocatechin, tetrachlorocatechin, protocatechin, 3-methylcatechin, 4-methylcatechin, 3-methoxycatechin, and 4-nitrocatechin [145]. Evangelista et al. have noticed that the creation of a chimeric protein via the fusion of *P. putida* chlorocatechin-1,2-dioxygenase with low-complexity domains derived from DEAD-box protein (Dhh1) allows one to obtain protein-rich condensate droplets in which the enzyme retains its structure, main biophysical properties, and enzymatic activity toward 4-chlorocatechin. Thus, this system represents a prototype of a microreactor with potential utility for bioremediation. The possible use of biomolecular microreactors is a great advantage for conducting and controlling site-specific reactions [18].

An interesting example of the discovery of new enzymes in strains that degrade PAHs is the work of Haq et al. These researchers isolated the bacterium *Bacillus subtilis* IH-1 from wastewater contaminated with petroleum products and cultivated this microbe. The isolated strain manifested an ability to effectively degrade PAHs and various wastewater pollutants, and the oxidation of the pollutants proved to be mediated by a lignin peroxidase produced by the obtained strain [155]. This outcome is quite unusual because enzymes decomposing lignin—and in parallel routing PAHs into catalytic chains—are found much more often in fungi.

Fungal enzymes that oxidize PAHs are less substrate-specific and may therefore be more effective at degrading mixtures of these chemical compounds [21]. For example, laccases, which are copper-dependent enzymes, are known for their broad substrate specificity and are reported to be highly effective in the degradation of highly persistent PAHs. Laccases of fungi represent a promising tool for PAH degradation also because of their high catalytic efficiency. Nevertheless, bacterial laccases have also been actively studied lately and are attractive due to their high thermal stability and tolerance to organic compounds [156].

In addition to bioremediation tasks, researchers have proposed to employ various bacterial oxygenases—that can catalyze various transformations of PAHs—in the industry for the more efficient and regioselective synthesis of desired monomers from aromatic compounds of biomass. One of the most complicated and fundamental reactions in organic synthesis is the regiospecific hydroxylation of an aromatic ring [157,158,159,160,161,162,163]. The capacity of oxygenases and oxidases to regiospecifically hydroxylate an aromatic ring or a side-chain substituent of an aromatic substrate paves the way to the biocatalytic synthesis of compounds that are difficult to obtain by organic synthesis [157,162,164,165].

For instance, the use of *cis*,*cis*-muconic acid as a chemical platform provides easy access to some monomers employed in the synthesis of commercial plastics. This compound is also a metabolic intermediate in the β-ketoadipic acid pathway of many bacteria; therefore, currently, the microbial production of this substance from abundant renewable resources is investigated through metabolic engineering. Wilbert et al. have assessed the suitability of the strain *Novosphingobium aromaticivorans* DSM12444 for the production of *cis*,*cis*-muconic acid from aromatic compounds of biomass [166]. In particular, two previously uncharacterized enzymes, protocatechuic acid decarboxylase and catechol-1,2-dioxygenase, were investigated, which are required for the conversion of aromatic metabolic intermediates into *cis*,*cis*-muconic acid. The ability of the strain *N. aromaticivorans* DSM12444 to stoichiometrically synthesize *cis*,*cis*-muconic acid from aromatic substances in biomass was demonstrated as well.

## 7. Conclusions

With the current pressure on nature, issues of environmental remediation come to the forefront, which leads to the involvement of an increasing number of ecologists, chemists, microbiologists, and enzymologists in this area. In this regard, research articles and reviews on PAH remediation have regularly appeared in the literature. In recent years, intensive bioremediation research has identified many aromatic-compound-degrading microorganisms [31,167]. For some microbes, the metabolic pathways of pollutant degradation have been characterized in detail [107,142,143,144] but not for all of them [93]. These studies have considerably expanded the existing knowledge about the biodegradation of aromatic compounds. Some of the described isolates can degrade several types of xenobiotics and have attracted much attention in research because contaminants are rarely found alone at contaminated sites and are typically a part of a mixture of compounds. In 2023–2024, a few reviews on enzymatic approaches to bioremediation [168,169,170] and on the role of individual bacterial genera in PAH degradation [171,172] were published. In an extensive review [173], authors were focused on the bioremediation of PAHs by various microorganisms, i.e., bacteria, fungi, and algae. A very extensive and detailed review by Vijayanand et al. covers many aspects regarding the current state of the field in bacterial bioremediation approaches for PAHs [174]. The variety of reviews in this interdisciplinary area allows us to cover a larger number of research articles and to highlight the versatility of this area, related to the following: the nuances of enzymatic degradation pathways and analysis of the catalytic activity of the enzymes involved in the degradation of PAHs; modern approaches of enzyme applications for bioremediation, such as immobilization as well as the engineering of enzymes with desired properties; and the usage of consortia of microorganisms, which, for example, have the ability to degrade different compounds or are capable of growing in extreme conditions, etc.

Despite researchers’ initial optimism, bioremediation has not become a panacea for environmental pollution, and many aspects of this technology’s application require further investigation and testing. The feasibility of using microorganisms and enzymes for the bioremediation of hydrocarbon derivatives depends on resistance of the microbes/enzymes to various conditions and on the versatility and flexibility of their substrate specificity. Therefore, there are still relevant and topical issues related to the degradation of pollutants present in low concentrations, present in extreme environments (for example, saline-alkaline soils or deserts), or present in mixtures with other compounds as well as the degradation of pollutants that may be toxic or can be metabolized to toxic intermediates [93]. Increasing the diversity of microorganisms available for use in the biodegradation of various PAHs and the creation of bacterial consortia based on already studied microorganisms to increase the efficiency of bioremediation, to improve the stability of the bacteria used, and to reduce the amount of toxic byproducts are key strategies for designing effective approaches to the remediation of hydrocarbon-contaminated soils and aquatic environments. Another important strategy is the search for new enzymes for bioremediation and their improvement via genetic engineering.

## Figures and Tables

**Table 1:** List of some PAH-degrading bacteria.

Bacteria	Sample Source	Registered Degradation of PAH Compound	Specific Features *	References
*Achromobacter aegrifaciens*	Crude-oil-contaminated seawater (Bizerte, Tunisia)	Chrysene	Production of biosurfactant	[91]
*Acinetobacter baumannii* INP1, *Pseudomonas taiwanensis* PYR1	PAH-contaminated estuarine wetlands (China)	Pyrene, indeno(1,2,3-cd)pyrene		[92]
*Aeribacillus pallidus* UCPS2, *Bacillus axarquiensis* UCPD1, *Bacillus siamensis* GHP76, *Bacillus subtilis* subsp. *inaquosorum* U277	Unkeshwar hot springs (India)	Anthracene, fluorene, phenanthrene, pyrene	Degradation of a PAH mixture at 50 °C within a consortium	[69]
*Arthrobacter* sp. YC-RL1	Petroleum-contaminated soil (Xingtai City, Hebei Province, China)	*p*-Xylene, naphthalene, phenanthrene, biphenyl, *p*-nitrophenol, and bisphenol A	Degrading a mixture of PAHs	[93]
*Bacillus* sp. ASDC2, *Burkholderia* sp. ASDC3, *Rhodococcus* sp. ASDC1	Polluted soil sediments (Amlakhadi Canal, Ankleshwar, India)	Chrysene	Higher efficiency of degradation in consortia	[94]
*Bacillus anthracis*, *B. cereus*, *B. mojavensis*, *B. subtilis*	Oil-contaminated sludge, soil, and sea water (Borg Al Arab City, Egypt)	Anthracene, α-naphthol, catechol		[95]
*Bacillus subtilis* SR1	Petroleum-contaminated rhizosphere soil	Benzo[*a*]pyrene	Resistant to the presence of several heavy metals	[96]
*Bacillus velezensis, Microbacterium schleiferi*, *Pseudomonas aeruginosa*, *Xanthomonas boreopolis*	Crude-oil-contaminated soil	Naphthalene, anthracene, acenaphthylene, fluorene, acenaphthene, phenanthrene, pyrene, benzo[*a*]pyrene	The ability to degrade PAHs present in crude oil; higher efficiency of degradation in consortia	[66]
*Bacillus licheniformis* MTCC 5514	Marine samples (India)	Anthracene	Production of biosurfactant	[86]
*Burkholderia cepacia* complex	Sludge samples	Benzo[*a*]pyrene	Production of biosurfactant	[97]
*Cobetia marina*, *Rhodococcus soli*, *Pseudoalteromonas agarivorans*	Sediment samples (Sinduri beach in Taean, Republic of Korea)	Naphthalene, phenanthrene, pyrene		[98]
*Cupriavidus* sp. MTS-7	Site of a former gas plant in Australia that has been contaminated for a long time	Benzo[*a*]pyrene	Ability to degrade PAHs across a wide pH range, especially acidic pH, and in the presence of low concentrations of Cu, Pb, Zn, and Cd	[99]
*Dietzia psychralcaliphila*	Sediments (Deception Island, Antarctica)	Phenanthrene		[9]
*Klebsiella michiganensis* EF4, *K. oxytoca* ETN19	PAH-contaminated farmland soil (Zhenjiang City, Jiangsu, China)	Phenanthrene	Higher efficiency of degradation in consortia	[100]
*K. pneumoniae* AWD5	Soil from automobile workshop (India)	Pyrene	Stimulate plant growth	[101,102]
*Microbacterium esteraromaticum*, *Ochrobactrum anthropi*, *Pseudomonas aeruginosa, Pseudomonas mendocina*, *Stenotrophomonas maltophilia*	Contaminated soil from oil refinery and a tyre waste dump site (India)	Naphthalene, fluorene, phenanthrene, benzo[*b*]fluoranthene	The ability to degrade PAHs present in crude oil; higher efficiency of degradation in consortia	[84]
*Pelagerythrobacter* sp. N7	Saline soil samples (Shanxi Province, China)	Phenanthrene	Resistant to the presence of elevated salt concentrations	[103]
*Pseudarthrobacter* sp. L1SW	Contaminated soil from petroleum refinery (China)	Phenanthrene	Stimulates plant growth; resistant to the presence of heavy metals (Ni, Zn, and Cr)	[104]
*Pseudomonas aeruginosa*	Garden soil	Phenanthrene, anthracene		[83]
*Rhodococcus opacus*	Petroleum-contaminated soil (Samara, Russia)	Phenol, catechol		[87,88]
*Rhodococcus rhodochrous* ATCC 21198	Commercially available from the American Type Culture Collection	Fluorene, phenanthrene, anthracene, pyrene	Degrading a mixture of PAHs	[105]
*Serratia marcescens* S2I7	Petroleum-contaminated soil (India)	Benzo[*a*]pyrene	Cadmium sustainability	[106]
*Sphingobium xenophagum* D43FB	Soil samples (South Shetland Islands, Antarctica)	Phenanthrene	Cadmium sustainability	[107]

* Specific features such as resistance to the presence of heavy metals, biosurfactant production, etc., emphasized in the literature. Structures of the aforementioned chemical compounds are represented in Figure 1.

## Data Availability

Data are available from N.A.K. upon request. Tel.: +7-(383)-363-5175, e-mail: nikita.kuznetsov@niboch.nsc.ru.
